# Methods and Benefits of Measuring Human-Centered Design in Global Health

**DOI:** 10.9745/GHSP-D-21-00207

**Published:** 2021-11-29

**Authors:** Cheryl Heller, Anne LaFond, Lakshmi Murthy

**Affiliations:** a The MeasureD Lab, New York, USA.; b John Snow Inc., Arlington, VA, USA.; c Jatan Sansthan, Udaipur, India.

## Abstract

Monitoring and evaluation (M&E), a new frontier for human-centered design (HCD), is still largely unexplored. In global health, M&E is considered essential to good practice, and evidence and data are critical tools in program design, performance monitoring, impact evaluation, and adaptation and learning. As HCD is increasingly integrated into global health practice, designers and global health practitioners are learning as they go how to integrate measurement into design and adapt traditional M&E approaches to design-influenced global health projects. This article illustrates some of the tensions inherent in the way global health and HCD practitioners approach measurement, using several cases to illustrate the ways in which tensions can be managed. Using framing introduced by the MeasureD project, which aimed to audit measurement practices in HCD (called social design in the MeasureD project), we explore 3 recent examples of design-influenced global health interventions: 1 focusing on products, 1 on behavior change, and 1 on service improvement, to extract learning about how teams used measurement, for what purpose, and to what effect. In comparing these examples and recent experience, we report on the steps being taken toward greater alignment in the use of measurement to advance human-centered global health programming.

## Abstract (precis)

Human-centered design practitioners should not overlook the value of systematic and standardized documentation and measurement inherent in global health and should consider ways to link design insights and solution development processes to traditional global health outcome and impact measures.

## Key Messages

- Integrating human-centered design (HCD) and global health requires new approaches to managing measurement across multidisciplinary project teams that:
  - Optimize the rigor of public health monitoring and evaluation
  - Introduce appropriate measurement into creative HCD approaches without compromising the types of learning that HCD can uniquely provide
- Tensions can emerge in the ways that HCD and global health approach measurement, but these can be addressed by appreciating the value of iterative learning, combining measurement approaches, and increasing transparency and documentation.
- To be relevant and effective in HCD-influenced health programming, global health practitioners and evaluators should adapt the timing and approach of traditional measurement approaches, integrate metrics that reflect user experience and desires, and use methods that facilitate adaptation and learning as well as assess performance and impact.

## INTRODUCTION

Strategies to measure and evaluate efforts to improve human health in low-income settings are well established and documented.^1^ However, measurement of human-centered design (HCD) processes and the use of measurement in design-influenced global health programming are still a new frontier.^2^ As HCD is increasingly integrated into global health practice, designers and global health practitioners are learning how to use measurement effectively during design, adapt traditional public health monitoring and evaluation (M&E) approaches to design-influenced projects, and assess the influence of design on global health program processes and outcomes. HCD has been introduced to global health interventions in various ways, but current understanding about when and how to use measurement and even what value is derived from measuring is incomplete.

Health programmers and evaluators experience frustration when integrating public health measurement approaches into HCD-led programs. Accustomed to standards that reflect rigor and evidence, they anticipate scores or metrics to describe insights or behavioral choices emerging from design research and solution prototyping, raising questions about sample size and respondent segmentation. Impact evaluation approaches in design-led projects are frequently derailed or delayed by the iterative nature of the design process. Rigorous mixed methods studies are not always responsive to the rapid learning pace of design. To date, there is no handbook of the M&E of HCD in global health that articulates an evaluation approach or framework that is fit for purpose and embraced by both design and health practitioners.

In this article, we build on an investigation begun in 2017, with support from the Robert Wood Johnson Foundation, that set out to audit measurement practices in HCD in a variety of initiatives. For the MeasureD project,^3^ we identified 31 initiatives from 27 different organizations to understand common methods and patterns of measurement, then developed cases for 8 projects. As part of our learning, we identified a framework consisting of 4 distinct ways in which measurement can be integrated into interventions that apply HCD, along with the types of learning that accrue from each. We began this review with those same questions, building on them to explore the experience of measurement in the context of HCD-influenced health programming.

We identified a framework consisting of 4 distinct ways in which measurement can be integrated into interventions that apply HCD, along with the types of learning that accrue from each.

We chose 3 global health cases, for diversity of project maturity and the amount of HCD and measurement involved, for this article (Table). A360, a large-scale, 3-country initiative, is an example of a comprehensive and strategic approach, both in terms of integration of HCD in adolescent sexual and reproductive health and in the investment made in documentation and measurement. The Brilliance series of products from Equalize Health provide an opportunity to observe measurement as it is integrated into a more traditional HCD process—to develop and deliver medical devices over a period of time and through multiple iterations based on continual learning. Group ANC, a small project from Scope Impact, is a useful example of early investment in measurement and experimentation in the role as well as the methods of measurement. We provide an overview of each project, the role played by HCD, and approaches to measurement, followed by a discussion of the accumulated lessons from these and other experiences.

**TABLE.** Three Examples of Using Measurement in Human-Centered Design-Influenced Health Interventions

**Project**	**Measurement Approach**
**Adolescents 360,** 2016–2020 Focus: Behavior change and service improvement Mechanism: Multi-partner collaboration An adolescent sexual and reproductive health program intended to increase demand for and voluntary uptake of modern contraceptives among adolescent girls aged 15–19 years in Ethiopia, Nigeria, and Tanzania	Clear problem statement and theory of changeUser-centered design researchIterative solution framingProcess evaluation including interviews, document review, and observationContinued measurement and iteration during implementationTheory-based independent outcome evaluation Cost effectiveness study
**Brilliance**, 2010–presentFocus: Product designMechanism: Single, multi-disciplinary organizationA line of phototherapy devices intended to close the quality health care gap for underserved newborns requiring jaundice management	Clear problem statement and theory of changeLandscape analysisUser-centered design researchInterviews with medical professionalsClinical observationHuman factors testing, theory of changeCustomer value chain analysisSupply chain analysisAdaptive management: routine monitoring and cycles of product adaptation
**Group Antenatal Care**, 2016–2019Focus: Service improvement and behavior changeMechanism: Multi-partner collaborationDevelop and test group ANC models as alternatives for addressing shortcomings of one-to-one models to deliver higher-quality care	User-centered design researchCo-creation of potential models with key stakeholders including mock pregnancy club sessions, card sorting, and testing of visuals, focused discussionsRoutine data analysisInterviews, focus group discussions, and observation of ANC pre/postTime and motion studiesMaterials testing

Abbreviation: ANC, antenatal care.

## OBSERVATIONS ON MEASUREMENT WHEN INTEGRATING HCD IN GLOBAL HEALTH

### Adolescents 360

Adolescents 360,^4^ an adolescent sexual and reproductive health program, integrated HCD to increase the demand for and voluntary uptake of modern contraceptives among adolescent girls aged 15–19 years in Ethiopia, Nigeria, and Tanzania. It was implemented from 2016 to 2020 by Population Services International in collaboration with IDEO.org as the HCD partner. In addition to design research and iterative solution framing during HCD, the project made a comprehensive investment in measurement and evaluation led by ITAD and in collaboration with the London School of Hygiene and Tropical Medicine and Avenir Health, including a theory-based independent evaluation with 3 core components: cost-effectiveness, outcome, and process evaluations. A360 generated several lessons about how to integrate design-led measurement and learning with traditional public health M&E.

First, the A360 team used HCD-generated insights to gain greater understanding of adolescents’ experience and desires related to relationships, health, and future livelihoods to help the project address its value proposition: to help girls understand the relevance and value of contraception to their lives. From this baseline understanding of the user, they crafted process documentation and monitoring tools such as user journey monitoring to track the project effectiveness over time using the girls’ framing of their desired experience when seeking contraceptive services. The A360 team learned that in an HCD-led intervention, the primary purpose of assessment and learning is not only measuring program outcomes (e.g., contraceptive adoption and continuation) but also ensuring fidelity to the idea of enabling girls to take their preferred journey toward decision and action related to contraceptive use. Process evaluation was found to be well-suited to this phase of a project that integrates HCD. As an A360 manager noted:

*In HCD, the user journey is deeply tied to the unique value proposition that you are trying to offer. The whole idea of HCD interventions is you are trying to offer a different user experience than they had before, one that is centered on their experience…… and you must monitor your ability to [maintain] fidelity to that user experience.*

The deliberate orientation of routine monitoring toward human-centered elements of the intervention helped the team “learn things that we did not know we needed to learn” to assess program performance. As an A360 program manager noted:

*These were not typical public health metrics or outcomes, but the whole success of the intervention hinged on this.*

Second, the team learned the hard way that the evaluation strategy designed to deliver a rigorous assessment of the impact of the intervention was introduced too early in the lifespan of the intervention. The impact evaluation was designed based on the broad program strategy, and baseline measures taken before intervention strategies and sites were defined and refined through design research and prototyping of solutions. The program design emerged much later than normal leading to a misalignment of evaluation and implementation strategies.

Third, on a positive note, the use of early-stage iteration and adaption of prototypes that is inherent in HCD encouraged continued iteration and optimization of the full-scale interventions beyond the design stage. The evaluation and implementation teams together reviewed data routinely to decide on the necessity of course corrections and to support real-time problem-solving. This collaboration on measurement and reflection ensured that the HCD-designed interventions delivered the value intended for girls and helped implementers know where and why to adapt once the design phase ended.

Collaboration on measurement and reflection ensured that the HCD-designed interventions delivered the value intended.

### Brilliance

Brilliance, introduced in 2010, is a phototherapy device intended to close the quality health care gap for underserved newborns requiring jaundice management. Unlike A360, which brought together several diverse partner organizations, Equalize Health is a single, multidisciplinary organization that integrates design and engineering and partners with medical and business professionals at various points along the process of design, development, and product distribution. Brilliance is one of Equalize Health’s earliest projects and has become a benchmark in the design for impact space.^5^ While the outcomes attributable to Brilliance are impressive, we focus on the seamless integration of HCD measurement with traditional monitoring and evaluation. Over the last 10 years, Equalize Health has continually tested and improved the adoption and impact of Brilliance, using an iterative process of HCD and adaptive management. The result of this is a series of new product variations, based on specific user needs and feedback, that have contributed to the growth, delivery, and impact of the Brilliance line.

Brilliance (classic) was developed in response to doctors in India and Nigeria lamenting the very low (5%–10%) prevalence of effective phototherapy devices in those countries. Through initial user-centered research, Equalize Health discovered that (1) existing phototherapy devices were too challenging to deliver and maintain, and (2) the incandescent or fluorescent bulbs that most of them used burned out every 6 months and were costly to replace. Once Brilliance was in the market, interviews with doctors uncovered the need for an “underside” version for when light was desired to reach the back and front of the baby. The result was Brilliance Underside, launched in 2012. Additional feedback from customers, partners, and in-hospital staff led to the introduction of Brilliance Pro in 2014, with significantly improved usability: thinner, lighter, easier to tilt with 1 hand, 2 treatment settings, and new technology that adjusted irradiance levels to be consistent across the baby’s skin. In 2015, an inexpensive light meter was introduced (the Brilliance Pro Light Meter) to allow nurses and doctors to measure irradiance themselves and be assured that they were providing correct dosage.

Each of these improvements was driven by evidence emerging from Equalize Health’s HCD approach and illustrates the benefits that measurement during HCD can contribute through discovery of nuanced user needs that might be missed by traditional methods. For example, the technology for phototherapy—even LED phototherapy—existed before Brilliance was introduced. Through an HCD process of discovery, Equalize Health listened to clinicians whom other device manufacturers ignored and solved problems for these individuals’ specific needs.

Each improvement to Brilliance illustrates the benefits that measurement during HCD can contribute through discovery of nuanced user needs that might be missed by traditional methods.

Equalize Health used several tools for measurement, including a clear problem statement, stakeholder surveys, clinical observation, landscape analysis, human factors testing, theory of change, customer value chain analysis, and pairwise comparison ranking (for device features as well as partner characteristics). After Brilliance Classic was launched, measurement, in the form of customer surveys, clinical observation, and monitoring was also used as part of the intervention to assess whether the device was reaching target customers, whether it was being used as intended, and whether improvements could be made for increased impact. Methods included monitoring sales, installations, and feedback from customers and the commercial partner, collecting demographic data about the hospitals, interviewing users about their experience, conducting surveys around clinician experience and preferences, and collecting data directly from the Brilliance devices to measure frequency of use. In cases where usage was lower than expected, Equalize Health interviewed clinicians and administrators to understand why— illustrating an important attribute of HCD measurement that is often neglected in global health M&E: to garner nuanced understanding that can contribute to improvements. Use of measurement and learning during the process of product development and supply chain mapping ensured effectiveness for users and made it possible for 1,111,300 babies to be treated with Brilliance as of January 2021.

### Group Antenatal Care

The group antenatal care (ANC) model was contextually developed in a partnership between the design agency Scope Impact and Management Sciences for Health. Group ANC models that promote self-care and social support have emerged as a promising alternative for addressing shortcomings of one-to-one models and delivering quality care.^6^^,^^7^ Group ANC was designed for communities in Uganda and Kenya and adapted to improve ANC services in Guatemala. The premise of this initiative is that ANC services are often not designed to meet women’s needs, resulting in negative experiences that can discourage engagement. Since the degree to which women engage with ANC is dependent on their level of trust and the quality of their experience, an HCD approach was used to understand client and care provider needs and preferences and to adapt services and materials to local contexts. The program consisted of a concept design and feasibility study in Uganda in 2016, a pilot study in Kenya in 2017, and adaptation and pilot in Guatemala in 2019. In each context, HCD was used to understand and incorporate both women’s and providers’ perspectives.

An initial discovery phase was followed by a co-creation phase with key stakeholders, including mock pregnancy club sessions, card sorting, and testing of visuals, focused discussions about challenges, support materials, schedule management and logistics of travel and transportation. In Uganda, 1 cohort of women completed the program, and the team conducted qualitative research to understand women’s experience of group care. Learning from Uganda was applied to models in Kenya: for example, appointments were scheduled in the afternoon to mitigate logistical issues that were observed among users in Uganda. In Kenya, 22 groups with approximately 12 women per group were established in 6 sites. Implementation in Kenya lasted approximately 1 year. Routine data from 1,090 women were analyzed to follow retention. The team conducted interviews, focus group discussions, and observation of ANC before and after the introduction of pregnancy clubs to understand feelings of agency, degree of satisfaction, and overall ratings of quality in care. They also conducted time and motion studies and tested materials with mothers and health care workers.

Despite limitations of time and money, the team acquired valuable learning, specifically questions about trust, connection, empowerment, and participants’ ability to implement the advice they received in the group ANC model. Importantly, they not only monitored the effect of the new model, but also were able to understand why it was or was not working, which provided them the opportunity to adapt their approach.

The Group ANC team not only monitored the effect of the new model, but also were able to understand why it was or was not working, which provided them the opportunity to adapt their approach.

As of 2019, learning to date is being piloted in 10 facilities in Mayan communities in Guatemala and will be scaled up to 30 health facilities over the next 2 years.

## TENSIONS THAT EMERGE DURING MEASUREMENT

From these cases and other efforts to explore measurement in the context of integration of HCD and global health practice,^2^^,^^8^^,^^9^ we observed several common tensions that emerge when both disciplines collaborate in the collection and use of data and evidence. These tensions relate to: (1) use of data for problem framing and intervention design, (2) the role of measurement, (3) the cadence and timing of measurement, (4) perceptions of rigor in measurement, and (5) documentation and transparency. We discuss the ways in which these tensions appear and the solutions evidenced in the cases reviewed for this article.

### Use of Data in Problem Framing and Intervention Design

Global public health interventions focus on an overarching goal: to improve human health. When HCD is applied to project planning, designers introduce an open and creative mindset and place understanding of human desires, behavior, and experiences at the forefront of design decisions. HCD principles hold that until context, including nuanced user needs and attitudes, is fully understood, the problem to be solved cannot be accurately defined. Also, HCD holds that iterative, small steps that incorporate user feedback lead to more reliable solutions. Reframing problems and iterating solutions begin early and may happen several times before defining the intervention, and the user (i.e., client, provider, or community) has agency in shaping solutions. These HCD principles require a comfort level with ambiguity, flexible research methods, and iterative learning that can be difficult for nondesigners to embrace.^10^

HCD holds that iterative, small steps that incorporate user feedback lead to more reliable solutions.

In contrast, traditional health project development often occurs in the context of procurement processes required to generate resources to execute solutions, such as national planning, budgeting, and competitive contracting or grant making. In these cases, problems to be addressed are mostly predefined by government or funding partners and solution choices are limited to a range of options based on a generalized or localized body of experience and science. The project design phase is embedded in proposal development, taking place over a short time, away from the sites and people who will engage with and benefit from the intervention. Co-creation or collaboration with those people and adaptation and tailoring of solutions to different communities or contexts following a funding decision (e.g., grant, contract, or budget) are often not encouraged. This kind of approach to health intervention design places future bets for success on existing evidence and past experience and favors technical expertise in solution development over new contextual learning.

### The Role of Measurement

The different ways in which designers and health program planners approach problem solving is reflected in their use of measurement to inform and guide their work. HCD takes a “make to learn” approach that integrates the processes of gathering data, making small experiments (prototypes), and generating learning to discover users’ needs, aspirations, and attitudes. HCD then tests solutions through several common methods that elicit user feedback in real time. The focus of measurement is learning that helps define and refine solutions to enhance their relevance to people, communities, workplaces, and systems.^11^ Measurement in HCD processes is rarely used to assess the overall effectiveness of an intervention or to track change over time.

Compared to HCD, global health devotes considerable professional and financial resources to measurement (e.g., research, monitoring, evaluation, assessment and increasingly predictive analytics and modeling) at all stages of programming. Use of evidence to inform intervention design and M&E to assess intervention effects are considered essential to good practice. Program implementers apply measurement to assess the status of conditions and define problems (e.g., needs assessments and baseline surveys) and to assess changes in these conditions (e.g., evaluation) to understand or prove the cause-and-effect link between interventions and intended outcomes. In this process, they assess variables such as uptake, use, access, quality, and health status, as well as cost effectiveness and sustainability. The focus is largely learning for tracking performance, testing solutions, and accountability once the intervention is launched, with limited investment in the use of evidence to develop program theory or inform intervention design.

### Cadence and Timing

When HCD is introduced into global health programming, its “make to learn” mindset often derails the steps and timing of traditional project implementation and measurement processes. In the HCD process, investigation takes place before problems are fully framed or root causes are identified. This conflicts with the traditional approach to health intervention design (e.g., proposal development and project planning), which often frames the problem and determines the solution before engaging clients and communities. An HCD approach requires a fluid use of measurement tools and strategies to surface user experience or perceptions to inform solution generation and program theory. In A360, designers worked with program implementers and service clients to evolve the program strategy and focus through user insights and testing solutions with clients after the project was planned and funded. At the same time, the program implementers and evaluators executed baseline measurement, but on reflection, as respondents reported, it would have been better to delay defining and gathering fully predictive baseline metrics until early-stage intervention shaping was complete because strategies, population groups, and intervention sites changed from the original proposal plan.

### Perceptions of Rigor in Measurement

A related tension emerges from program implementers who consider HCD measurement to be less rigorous than methods typically applied in health program M&E. Global health uses widely accepted approaches to applied research and program evaluation, which draws on public health, behavioral science, organizational strategies, and other disciplines. Credibility in measurement is derived from standardization, systematic approaches, and scientific logic using theories of change with well-formulated hypotheses of program strategy and applying methods that reflect the level of rigor required to produce reliable evidence for decision making. A program manager in A360 reported that she questioned the insight decks and prototype report cards produced during the design process because they lacked the nuance and precision related to segmentation of respondents by age, geography, and other characteristics that were expected in a public health program. In other examples, design research did not integrate process or outcome metrics which health program implementers find familiar and reliable nor did they articulate the link between design-generated solutions and health program outcomes and impacts.

Designers explain that rigor in the design process derives from the clear articulation of the objectives for learning and assiduous monitoring of progress toward stated objectives. As a rule, design researchers, like health researchers, map out data collection strategies (e.g., for formative purposes). They apply mainly qualitative approaches influenced by ethnography, psychology, and user-experience or service design mainly in the program design stage. In contrast to public health research, designers rely on small samples and short data-generation and analysis timeframes to provide faster feedback at interim stages of solution development. They also modify methods and lines of inquiry based on what they are learning, allowing opportunity to follow and confirm unanticipated discoveries that emerge.

HCD’s approach to measurement can appear to nondesigners as unstructured or unintentional, but it is inherently creative as well as strategic in its adherence to learning goals. Designers make the case that the “proof of concept” that results from incorporating user feedback along the way justifies the investment of time in the design process. Rather than sticking with an approach that is not producing desired results, designers use learning to refine ideas or pivot before large intervention investments are made. What may look like a lack of discipline is simply a different discipline, which public health implementers are only starting to integrate in a systematic way by using adaptive learning approaches.^12^ By the same token, designers can view attempts to monitor the process too closely or rigidly to gain insights from users as restrictive and detrimental. Theories of change and predefined metrics of success articulated at the proposal stage typically come in too early when applying HCD and may undermine the design process.

To nondesigners, HCD’s approach to measurement can appear to be unstructured or unintentional, but it is inherently creative as well as strategic in its adherence to learning goals.

### Documentation and Transparency

Finally, health program implementers can be frustrated by the limited transparency in the criteria that designers use to develop insights or determine when a prototype should move from low to high resolution. In the words of a team leader of an organization included in our case review, there is “no consistent methodology that captures both the tangible and intangible benefits of creative methods and solutions” nor is there agreement about how to capture the intangible decision-making criteria that drive design processes. The open, learning-focused process for finding working solutions in complex settings contrasts with the predefined, often rigid approaches to program development and implementation in global health.^13^

There have been recent calls for better documentation of HCD-led interventions to advance the evidence base which will help address this tension.^14^

## HOW TO ADDRESS TENSIONS

Within the emerging body of experimentation and collaboration, HCD and global health teams are beginning to merge their practices and find ways to address the discord and tension of early experiences. For example, they are integrating human-centered methods for studying user experience with segmentation studies to provide a comprehensive picture of communities and clients. They are also mapping user perspectives and experience gained through design research into pathways in theories of change, connecting design-led solutions to public health outcomes (e.g., uptake and continuation), and conducting impact evaluations to test and document the influence of HCD. Behavioral design, a process that integrates design and evidence-driven measurement,^15^ may also provide lessons on how to optimize different measurement approaches. Thus, while tensions around different approaches to programming and measurement can lead to disruptions and disconnect, as HCD and global health work together, each is adapting and aligning its approach and as a result, working to better overall effect. We discuss 3 specific ways of easing the tensions: appreciate iterative learning, combine measurement approaches, and increase transparency and documentation of HCD-related measurement and decision making.

### Appreciate Iterative Learning

An acknowledged benefit from design is the primacy of iteration fueled by reflection and learning. Just as the technology industry understands that any product or service needs to continually adjust based on user feedback and new learning, the same is true for health interventions. But whereas the technology industry accepts this notion of iteration as part of the process through which their offerings stay relevant, it is only beginning to find fertile ground in global health, especially within the constructs of funding agreements and expectation for performance monitoring and impact evaluation. A designer observed that public health does not easily accept failure and learning as a path to solution development. The evolution of the Brilliance phototherapy products is an example of the benefits of iteration. Through a continual program of human-centered research, the Equalize Health team discovered 2 deterrents to adoption of the product: first, that the cost of lightbulbs was beyond what many users could afford, and second, that some doctors were not using the product because they did not know how to treat jaundice. In both cases, with human-centered approaches to learning, the team was able to discover and address these unanticipated issues and increase product adoption. When projects move from the prototyping phase to full-scale implementation and HCD-led intervention choices are translated into action, practitioners are often inspired to continue iterating with measurement. HCD models of iterative intervention testing are helpful to actors seeking to integrate collaboration, learning, and adaptation (CLA)^16^ approaches advocated by the U.S. Agency for International Development and supported by numerous development movements (e.g., Doing Development Differently Manifesto^17^ and Thinking and Working Politically^18^).

A designer observed that public health does not easily accept failure and learning as a path to solution development.

### Combine Measurement Approaches

Our review found that many health and design projects are able to blend research and measurement strategies, drawing from both disciplines to optimize learning. In moving from the design phase and adopting an HCD-iterative approach to implementation, both Equalize Health and Population Services International in A360 used both traditional quantitative approaches (e.g., client exit interview surveys) and qualitative HCD measurement (e.g., journey mapping) to understand client experience. With respect to unpacking the response of younger girls in Nigeria to service messaging and offerings, a A360 program manager noted:

*Lean quantitative and heavy qualitative [approaches] helped us see things we had a hard time explaining.*

Based on learning from various data sources, they adjusted the fit between their service delivery and client needs to tailor the program to specific user groups (e.g., A360) or ensure the sustained availability and affordability of a medical device.

In a second example of blended approaches, health program implementers are incorporating “human-centered metrics” into traditional M&E plans. These HCD-metrics describe user needs and desires uncovered in the early design phase which represent intermediate steps toward outcomes and impact. In A360, program managers discovered that the “true North in design interventions is not necessarily fidelity to the public health intervention as a whole but to the user experience of that intervention as a critical driver of success.”

HCD focuses public health practitioners on the user experience as an intermediate step toward public health outcomes such as uptake or coverage that are inherently linked to human perceptions, desires, behavioral drivers, and satisfaction (or delight). Success is defined by whether users embrace and engage with an intervention as well as whether an intervention results in greater service uptake or continuity.

### Increase Transparency and Documentation

To improve understanding of HCD approaches across health and design teams and address concerns about the rigor of HCD measurement, an implementer of one of our cases introduced detailed documentation of the methods and findings that informed design research and decision criteria that were used to select prototypes for further testing and refinement. They created detailed summaries of insights gathered during the design research^19^ and prototype report cards^20^ to help team members, stakeholders, and funders understand the learning from HCD and the refinement of solutions. This and other steps toward greater transparency and accountability across the team helped health program managers understand and accept design methods, increase the potential for replicability of findings and reduce the potential for uncertainty and doubt. Striking a balance between the need for rigor and the value of creativity remains a challenge in these kinds of projects. Most respondents noted that if rigorous measurement is imposed on HCD at the wrong time, it risks “taking the creative spark out of HCD.” As a program manager noted:

*Managing HCD requires you to take the foot off the evidence and rigor because it does something to the creativity.*

Striking a balance between the need for rigor and the value of creativity remains a challenge in these kinds of projects.

## CONCLUSIONS

Measurement in the context of HCD-led global health programming is an evolving practice. While tensions around measurement can be confusing or even vexing to those involved, learning suggests that there are practical ways to alleviate these tensions and optimize the application of measurement when HCD is applied in global health. Based on our observations from these case examples and consultation with design and global health practitioners, we posit that increased understanding of how measurement strategies differ and complement one another will benefit global health interventions overall and optimize the influence of HCD in this context. While measurement conducted during HCD is not a substitute for surveys, evaluations, and research typical of public health programming, it is vital to providing insights that help define and deliver appropriate and sustainable products, services, and interventions. Measuring in the context of HCD can provide different and important learning that is additive and can be critical to risk reduction. To work effectively with design, traditional public health M&E framing and methods require adaptation to the rhythm and process of HCD interventions. Global health evaluators can also consider HCD-generated metrics related to user desires and experience for tracking program effectiveness.

## Notes

**Peer Reviewed**
